# Supplementary material for: Primary Series and Booster Coronavirus Disease 2019 Vaccine Effectiveness in a Cohort of Healthcare Workers in Albania During a BA.1 and BA.2 Variant Period, January–May 2022
Source: Open Forum Infect Dis. 2023 Oct 12;10(10):ofad479. doi: 10.1093/ofid/ofad479 (PMC10599317; doi:10.1093/ofid/ofad479)
Supplement: ofad479_Supplementary_Data [file ofad479_supplementary_data.docx]

**Supplemental information**

**Primary series and booster COVID-19 vaccine effectiveness in a cohort of healthcare workers in Albania during a BA.1 and BA.2 variant period, January – May 2022**

***Methods***

*COVID-19 case definition*

During the study, participants completed weekly symptom questionnaires; participants who reported having any symptom included in the Albanian MOH COVID-19 case definition (fever, cough, general weakness, fatigue, headache, muscle aches, sore throat, runny nose, shortness of breath, lack of appetite, nausea, vomiting, diarrhoea, altered mental state, loss of taste, loss of smell).

*Sensitivity analyses*

We also performed two sensitivity analyses: i) we changed the definition of “time at risk” from 90 days after infection to 60 days after a positive PCR or RAT test, assuming a shorter duration of protection from infection; ii) we considered participants “fully immunised” 7 days, rather than 14 days, after their second or third dose.

***Results***

*Sensitivity analyses*

In the two sensitivity analyses, where we i) modified the participant inclusion window after infection from 90 days to 60 days after infection and ii) where we considered participants fully immunized 7 days (rather than 14 days) after their last vaccine dose, the VE estimates changed by less than 10% (Table S3).


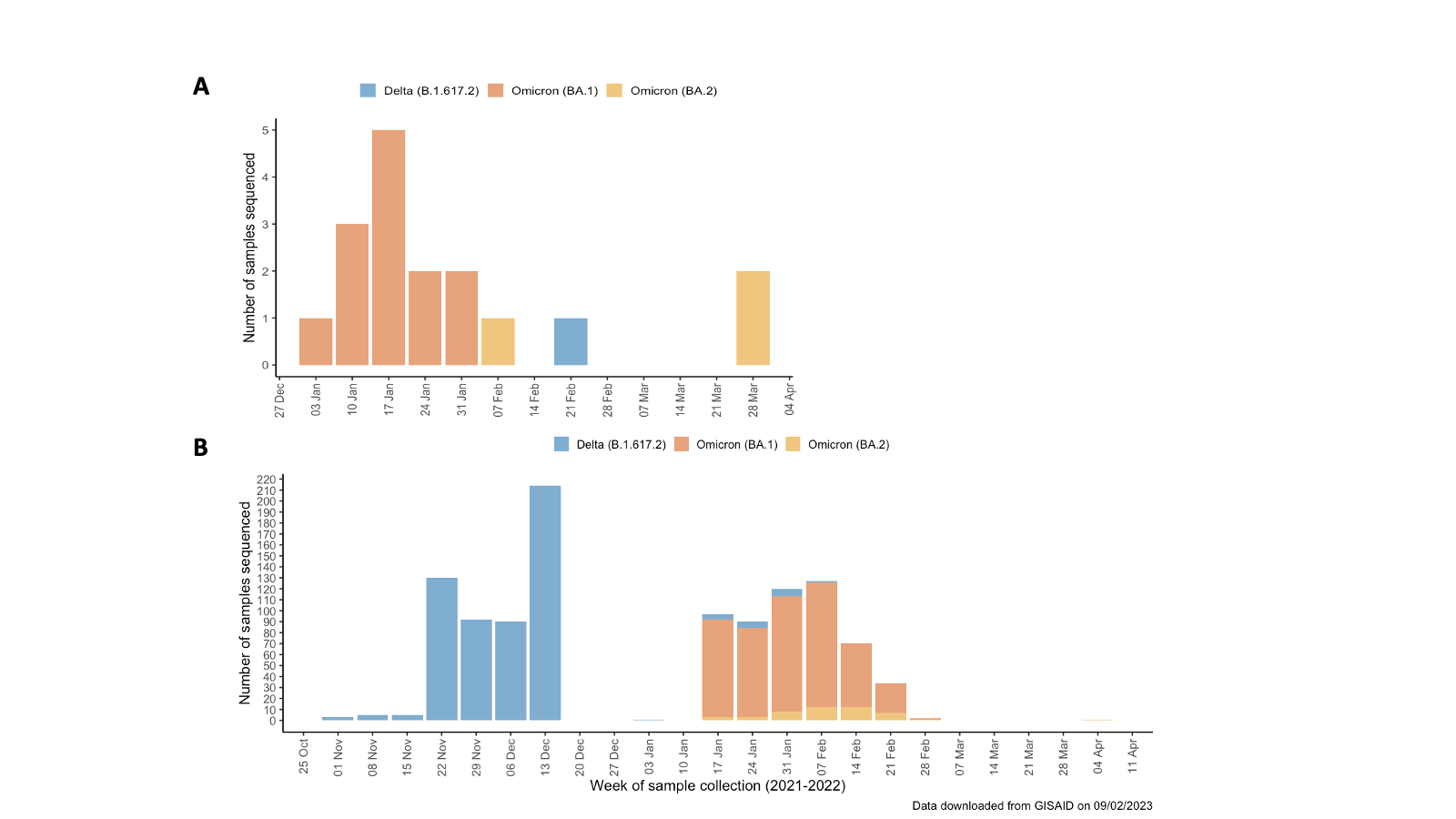


**Figure S1. Whole genome sequencing results (A) PCR-positive specimens from study participants in 2022 and (B) molecular surveillance data from Albania downloaded from GISAID from November 2021 to April 2022**

**
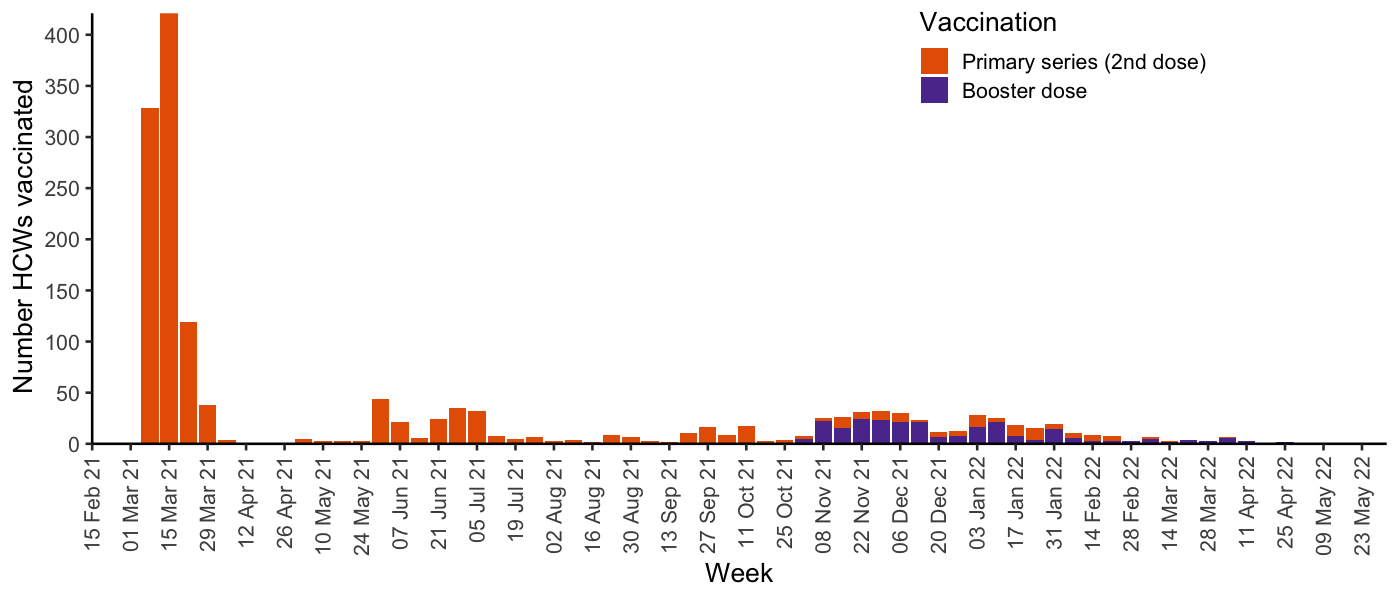
**

**Figure S2. Week of receipt of COVID-19 vaccine primary series 2^nd^ dose or booster dose in 2021 and 2022 among study participants**

**Table S1. Participant demographics and characteristics by vaccination status on the end day of follow-up (n=1462)**

|  | **All Participants** | **Unvaccinated** | **Received primary vaccine series** | **Received first booster dose** |
| --- | --- | --- | --- | --- |
| Age in years at end of person-time contribution | | | | |
| Median (IQR) | 44 (34-53) | 36 (30-46.8) | 43 (34-52) | 50 (41-56.5) |
| Age group in years at end of person-time contribution | | | | |
| 20-29, n (%) | 208 (14) | 21 (21) | 161 (15) | 15 (6) |
| 30-39, n (%) | 381 (26) | 38 (39) | 283 (26) | 38 (15) |
| 40-49, n (%) | 364 (25) | 16 (16) | 275 (26) | 69 (28) |
| 50-59, n (%) | 407 (28) | 19 (19) | 292 (27) | 87 (35) |
| 60+, n (%) | 102 (7) | 4 (4) | 59 (6) | 38 (15) |
| Sex | | | | |
| Male, n (%) | 311 (21) | 9 (9) | 205 (19) | 87 (35) |
| Female, n (%) | 1151 (79) | 89 (91) | 865 (81) | 160 (65) |
| Hospital | | | | |
| Tirana, n (%) | 905 (62) | 63 (64) | 644 (60) | 173 (70) |
| Durres, n (%) | 297 (20) | 12 (12) | 224 (21) | 53 (21) |
| Fier, n (%) | 260 (18) | 23 (23) | 202 (19) | 21 (9) |
| Any chronic condition at enrolment | | | | |
| No, n (%) | 1175 (80) | 84 (86) | 868 (81) | 181 (73) |
| Yes, n (%) | 287 (20) | 14 (14) | 202 (19) | 66 (27) |
| Number of chronic conditions at enrolment | | | | |
| 0, n (%) | 1175 (80) | 84 (86) | 868 (81) | 181 (73) |
| 1, n (%) | 219 (15) | 10 (10) | 157 (15) | 48 (19) |
| 2+, n (%) | 68 (5) | 4 (4) | 45 (4) | 18 (7) |
| Smoking status at enrolment | | | | |
| Currently smokes, n (%) | 202 (14) | 9 (9) | 138 (13) | 50 (20) |
| Never smokes, n (%) | 1192 (82) | 88 (90) | 888 (83) | 179 (72) |
| Previously smokes, n (%) | 68 (5) | 1 (1) | 44 (4) | 18 (7) |
| BMI groups at enrolment | | | | |
| Underweight or normal, n (%) | 608 (42) | 48 (49) | 458 (43) | 73 (30) |
| Overweight, n (%) | 587 (40) | 32 (33) | 426 (40) | 118 (48) |
| Obese, n (%) | 267 (18) | 18 (18) | 186 (17) | 56 (23) |
| Self-assessed health status at enrolment | | | | |
| Excellent, n (%) | 340 (23) | 9 (9) | 264 (25) | 59 (24) |
| Very good, n (%) | 808 (55) | 65 (66) | 577 (54) | 139 (56) |
| Good, n (%) | 185 (13) | 15 (15) | 139 (13) | 23 (9) |
| Fair, n (%) | 120 (8) | 8 (8) | 83 (8) | 26 (11) |
| Poor, n (%) | 9 (<1) | 1 (1) | 7 (<1) | 0 (0) |
| Occupation/Role in hospital at enrolment | | | | |
| Physician, n (%) | 297 (20) | 12 (12) | 163 (15) | 115 (47) |
| Nurse or Midwife, n (%) | 691 (47) | 46 (47) | 536 (50) | 93 (38) |
| Janitorial Staff or Food Worker, n (%) | 194 (13) | 11 (11) | 157 (15) | 21 (9) |
| Other, n (%) | 280 (19) | 29 (30) | 214 (20) | 18 (7) |
| Patient facing at enrolment | | | | |
| No, n (%) | 68 (5) | 10 (10) | 45 (4) | 9 (4) |
| Yes, n (%) | 1394 (95) | 88 (90) | 1025 (96) | 238 (96) |
| Hands on care at enrolment | | | | |
| No, n (%) | 582 (40) | 42 (43) | 430 (40) | 86 (35) |
| Yes, n (%) | 880 (60) | 56 (57) | 640 (60) | 161 (65) |
| Household size at enrolment (N missing data = 55) | | | | |
| 1-3, n (%) | 557 (40) | 38 (40) | 386 (38) | 113 (47) |
| 4-5, n (%) | 733 (52) | 50 (53) | 555 (54) | 109 (46) |
| 6+, n (%) | 117 (8) | 7 (7) | 88 (9) | 17 (7) |
| SARS CoV-2 Vaccine formulation at end of follow-up | | | | |
| Unvaccinated, n (%) | 98 (7) | 98 (100) | 0 (0) | 0 (0) |
| 1 dose BNT162b2, n (%) | 31 (2) | 0 (0) | 0 (0) | 0 (0) |
| 2 dose BNT162b2, n (%) | 927 (63) | 0 (0) | 926 (87) | 1 (<1) |
| 3 dose BNT162b2, n (%) | 227 (16) | 0 (0) | 0 (0) | 227 (92) |
| 2 dose BNT162b2+ 1 dose mRNA-1273, n (%) | 1 (<1) | 0 (0) | 0 (0) | 1 (<1) |
| 1 dose ChAdOx1-S, n (%) | 14 (<1) | 0 (0) | 0 (0) | 0 (0) |
| 2 dose ChAdOx1-S, n (%) | 125 (9) | 0 (0) | 125 (12) | 0 (0) |
| 2 dose ChAdOx1-S + 1 dose BNT162b2, n(%) | 15 (1) | 0 (0) | 0 (0) | 15 (6) |
| 1 dose CoronaVac, n (%) | 2 (<1) | 0 (0) | 0 (0) | 0 (0) |
| 2 dose CoronaVac, n (%) | 9 (<1) | 0 (0) | 9 (<1) | 0 (0) |
| 2 dose CoronaVac + 1 dose BNT162b2, n (%) | 2 (<1) | 0 (0) | 0 (0) | 2 (<1) |
| Heterologous vaccination, n (%) | 11 (<1) | 0 (0) | 10 (<1) | 1 (<1) |

**Table S2. Unadjusted and adjusted COVID-19 vaccine effectiveness estimates (VE) stratified by prior infection and by eligibility to receive booster dose**

**(**Due to low power in categories with PCR- or PCR- and RAT- confirmed infection, VE could only be calculated for persons with evidence of prior infection)

|  | | | **Number of HCWs** | **Total person-time (days)** | **PCR-confirmed infection** | **RAT-confirmed infection** | **Sero- conversion** | **All infections** | **VE (95% CI)** |
| --- | --- | --- | --- | --- | --- | --- | --- | --- | --- |
| **Primary vaccine series vaccine effectiveness all cohort by prior infection status** | | | | | | | | |  |
| **Symptomatic infection** | **PCR** | Unvaccinated with prior infection | 122 | 12114 | 8 |  |  | 8 | Ref |
|  |  | Primary series - any vaccine + prior infection | 1010 | 125100 | 55 |  |  | 55 | 17.7 (-83.1; 63) |
|  |  | Unvaccinated with prior infection | 122 | 12114 | 8 |  |  | 8 | Ref |
|  |  | Primary series - BNT162b2 + prior infection | 877 | 107831 | 52 |  |  | 52 | 8.3 (-103.8; 58.8) |
|  | **PCR and RAT** | Unvaccinated with prior infection | 122 | 12114 | 8 | 2 |  | 10 | Ref |
|  |  | Primary series - any vaccine + prior infection | 1010 | 125100 | 55 | 7 |  | 62 | 28.5 (-49.4; 65.8) |
|  |  | Unvaccinated with prior infection | 122 | 12114 | 8 | 2 |  | 10 | Ref |
|  |  | Primary series - BNT162b2 + prior infection | 877 | 107831 | 52 | 7 |  | 59 | 20.6 (-66; 62) |
| **Any infection** | | Unvaccinated without prior infection | 8 | 510 | 2 | 0 | 2 | 4 | Ref |
|  |  | Primary series - any vaccine + no prior infection | 112 | 9730 | 7 | 1 | 38 | 46 | 59 (-24.6; 86.5) |
|  |  | Unvaccinated withprior infection | 121 | 11592 | 8 | 3 | 4 | 15 | Ref |
|  |  | Primary series - any vaccine + no prior infection | 996 | 118795 | 56 | 10 | 44 | 110 | 7.5 (-65.7; 48.4) |
|  |  | Unvaccinated without prior infection | 8 | 510 | 2 | 0 | 2 | 4 | Ref |
|  |  | Primary series - BNT162b2 + no prior infection | 95 | 8636 | 5 | 1 | 30 | 36 | 61.1 (-25.7; 88) |
|  |  | Unvaccinated with prior infection | 121 | 11592 | 8 | 3 | 4 | 15 | Ref |
|  |  | Primary series - BNT162b2 + prior infection | 877 | 104164 | 54 | 9 | 36 | 99 | 4.2 (-72.4; 46.8) |
| **Primary vaccine series vaccine effectiveness - participants eligible for booster** | | | | | | | | |  |
| **Symptomatic infection** | **PCR** | Unvaccinated | 130 | 12736 | 10 |  |  | 10 |  |
|  |  | Primary series - any vaccine | 1110 | 131043 | 51 |  |  | 51 | 42.2 (-23.3; 72.9) |
|  |  | Unvaccinated | 130 | 12736 | 10 |  |  | 10 |  |
|  |  | Primary series - BNT162b2 | 955 | 113450 | 45 |  |  | 45 | 40.7 (-26.6; 72.2) |
|  | **PCR and RAT** | Unvaccinated | 130 | 12736 | 10 | 2 |  | 12 |  |
|  |  | Primary series - any vaccine | 1110 | 131043 | 51 | 8 |  | 59 | 45.7 (-10; 73.2) |
|  |  | Unvaccinated | 130 | 12736 | 10 | 2 |  | 12 |  |
|  |  | Primary series - BNT162b2 | 955 | 113450 | 45 | 8 |  | 53 | 43.9 (-13.9; 72.4) |
| **Any infection** | | Unvaccinated | 129 | 12102 | 10 | 3 | 6 | 19 |  |
|  |  | Primary series - any vaccine | 1083 | 120282 | 51 | 11 | 76 | 138 | 22.5 (-30.2; 53.9) |
|  |  | Unvaccinated | 129 | 12102 | 10 | 3 | 6 | 19 |  |
|  |  | Primary series - BNT162b2 | 946 | 106021 | 46 | 10 | 62 | 118 | 23 (-30.1; 54.5) |
| **Booster dose vaccine effectiveness by prior infection status** | | | | | | | | |  |
| **Any infection** | | Unvaccinated with prior infection | 121 | 11592 | 8 | 3 | 4 | 15 | Ref |
|  |  | Booster dose - any vaccine + prior infection | 194 | 22722 | 4 | 0 | 6 | 10 | 54.2 (-11.2; 81.1) |
|  |  | Unvaccinated with prior infection | 121 | 11592 | 8 | 3 | 4 | 15 | Ref |
|  |  | Booster dose - BNT162b2 + prior infection | 182 | 21534 | 4 | 0 | 6 | 10 | 73.4 (-2.8; 93.1) |

**S3. Sensitivity analyses COVID-19 vaccine effectiveness against symptomatic PCR-confirmed SARS-CoV-2 infection for full cohort, estimated for any vaccine**

|  | **N participants** | **Total person-time (days)** | **Symptomatic PCR-confirmed COVID-19 infection** | **VE (95% CI)** |
| --- | --- | --- | --- | --- |
| **Fully vaccinated at 7 days after vaccination, instead of 14 days** | | | |  |
| **Eligible for booster doses - Two doses** |  |  |  |  |
| Unvaccinated | 130 | 12736 | 10 | Ref |
| ≥7d from 2nd dose | 1110 | 131449 | 51 | 42.8 (-21.9; 73.2) |
| **Eligible for booster doses - Three doses** |  |  |  |  |
| Unvaccinated | 130 | 12736 | 10 | Ref |
| ≥7d from 3^nd^ dose | 240 | 29955 | 4 | 89 (42.6; 97.9) |
| **Reinfection definition: 60 days instead of 90 days** | | | |  |
| **Eligible for booster doses - Two doses** |  |  |  |  |
| Unvaccinated | 132 | 12864 | 11 | Ref |
| ≥14 days from 2^nd^ dose | 1110 | 131849 | 51 | 46.8 (-11; 74.5) |
| **Eligible for booster doses - Three doses** |  |  |  |  |
| Unvaccinated | 132 | 12864 | 11 | Ref |
| ≥14 days from 3^nd^ dose | 240 | 29309 | 4 | 88.7 (45.5; 97.7) |
